# Supplementary material for: Cathepsin B abundance, activity and microglial localisation in Alzheimer’s disease-Down syndrome and early onset Alzheimer’s disease; the role of elevated cystatin B
Source: Acta Neuropathol Commun. 2023 Aug 14;11:132. doi: 10.1186/s40478-023-01632-8 (PMC10426223; doi:10.1186/s40478-023-01632-8)
Supplement: Supplementary file 1 — Additional file 1. Supplementary Figure 1: changes to cathepsin B and cathepsin D activity in a sub analysis of EOAD and AD-DS Braak VI cases. [file 40478_2023_1632_MOESM1_ESM.pdf]

Wu et al.,

Cathepsin B abundance, activity and microglial localisation in Alzheimer's disease – Down syndrome and early onset Alzheimer's disease; the role of elevated cystatin B

Supplementary Data

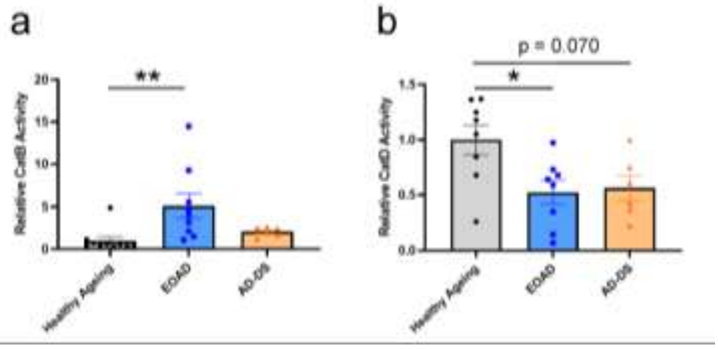

Supplementary Figure 1 Changes to cathepsin B and cathepsin D activity in a sub analysis of EOAD and AD-DS Braak VI cases

Sub-analysis of cathepsin B (**a**) and (**b**) cathepsin D activity in EOAD and AD-DS (Braak neurofibrillary tangle stage VI), and healthy-ageing. (**a**) Type of case affected cathepsin B activity (ANOVA $F(2,17) = 6.792$, $p = 0.007$); activity was significantly higher in individuals who had EOAD than in controls (pairwise comparisons with Bonferroni correction $p = 0.008$), with no difference between individuals with AD-DS and control individuals (pairwise comparisons with Bonferroni correction $p = 1.000$) or AD-DS and EOAD (pairwise comparisons with Bonferroni correction $p = 0.115$). There was a trend that the age at the time of death (in years) has an impact on activity (ANOVA $F (1,17) = 4.358$, $p = 0.053$) (Healthy ageing n = 9, EOAD n = 9, AD-DS n = 6). (**b**) Type of case (ANOVA $F(2,14) = 7.188$, $p = 0.007$) and age at death (ANOVA $F(2,14) = 5.214$, $p = 0.039$) significantly affected cathepsin D activity. Activity was significantly lower in individuals who had EOAD than in healthy controls (pairwise comparisons with Bonferroni correction $p = 0.028$), a trend for reduced activity was observed between AD-DS and healthy ageing (pairwise comparisons with Bonferroni correction, $p = 0.070$), with no difference in the activity between individuals with AD-DS and those with EOAD (pairwise comparisons $p = 1.000$) (Healthy ageing n = 8, EOAD n = 8 , AD-DS n = 6). Individual data points are technical means for independent biological samples, error bars SEM. * $p < 0.05$ and ** $p < 0.01$.
